# A case report on the treatment of diaphragmatic eventration-induced Budd-Chiari syndrome: the first innovative application of diaphragm plication

**DOI:** 10.3389/fmed.2025.1453967

**Published:** 2025-05-27

**Authors:** Jiming Ma, Bingjun Tang, Xuedong Wang, Liuqing Yang, Yumei Li, Yan Wen, Pengfei Wang

**Affiliations:** ^1^Beijing Tsinghua Changgung Hospital, Tsinghua University, Beijing, China; ^2^Xiang Yang No.1 People’s Hospital, Xiangyang, China

**Keywords:** Budd-Chiari syndrome, diaphragmatic eventration, diagnosis, diaphragmatic plication, case report

## Abstract

**Introduction:**

Budd-Chiari syndrome (BCS) is a condition in which the hepatic outflow tract is obstructed. In some rare situations, abnormal diaphragmatic eventration (DE) is the main cause of BCS; furthermore, severe cases of DE-induced BCS can lead to liver congestion, functional impairment, and cirrhosis. Only three cases of DE-induced BCS have been reported in the literature to date, and no specific treatment modalities have been described. Herein, we present the first case in which minimally invasive surgery was used to treat the DE-induced BCS.

**Case report:**

A 72 years-old woman presented with hepatic encephalopathy as the first symptom and a diagnosis of cirrhosis. Contrast-enhanced computed tomography (CT) and abdominal ultrasound imaging revealed obstruction of the hepatic outflow tract, stenosis of the hepatic vein (HV) at the opening of the inferior vena cava (IVC) and dilatation of the lower segment of the IVC. These findings were consistent with a diagnosis of BCS. After three interventional balloon dilatations, the stenosis could not be resolved. Chest radiographs revealed a marked elevation of the right diaphragm (by approximately three vertebral levels) compared with the left diaphragm. During the thoracic surgery consultation, DE was diagnosed as a possible underlying cause of BCS, and diaphragmatic plication (DP) was proposed as a surgical treatment. The patient then underwent DP at our center and was discharged on postoperative day 4 without complications. The follow-up examinations revealed that the patient’s blood ammonia level and internal diameter of the IVC had returned to normal.

**Conclusion:**

Diaphragmatic eventration is an exceedingly rare cause of BCS, and DP offers novel therapeutic insights for such condition. Etiology-driven strategies and appropriate multidisciplinary modalities are extremely necessary for diagnose and treat BCS.

## Introduction

Budd-Chiari syndrome (BCS) is caused by the obstruction of the hepatic outflow tracts (1). In some cases, this condition can cause recurrent abdominal pain and bloating of the right upper abdomen, ascites, jaundice, liver bruising and enlargement. Furthermore, severe BCS can progress to cirrhosis and eventually to liver failure (2). The etiology of the disease can be broadly classified as primary, lesions in the hepatic vein (HV) or inferior vena cava (IVC) associated with structural narrowing of the veins, thrombosis due to abnormal coagulation processes, and thromboembolic obstruction due to malignancy (3). External compression of the IVC, such as compression by solid tumors and abdominal organs through hernia formation, is the secondary cause of BCS (3). Diaphragmatic hernia (DH), which is relatively rare, can also contribute to this condition (4). Some of these contents, including the colon, the omentum or even part of the liver lobe, may herniate due to congenital weaknesses in the development of the diaphragm (5). A small percentage of diaphragmatic injuries are caused by trauma, which can compress the outflow tract by hernia formation and reduce the diameter of the IVC due to diaphragmatic scar healing and fibrotic tissue contracture (4).

Diaphragmatic eventration (DE) is a congenital developmental defect in the muscular part of the diaphragm that only remains attached to the sternum, ribs and dorsal lumbar vertebrae (6). According to embryological theory, structural defects in the diaphragm are caused by abnormal or delayed migration of myofibroblasts in the upper cervical segment (7, 8). DE is commonly induced by diaphragmatic paralysis that is caused by tumor- or trauma-mediated damage to the phrenic nerve (9, 10). Dyspnea is the most prominent clinical manifestation in patients with DE, and ventilation/perfusion mismatch and loss of chest wall compliance are among the factors that contribute to dyspnea. Some patients develop mild hypoxemia and attempt to compensate by hyperventilation, which can result in mild respiratory alkalosis (6). Atypical gastrointestinal symptoms may also occur in a subset of patients (especially those with left hemidiaphragm eventration), such as epigastric pain, bloating, heartburn, regurgitation, belching, nausea, constipation, and inability to gain weight (8, 11). However, DE-induced BCS is much rarer. Only three cases have been reported in the literature to date by Saujani etal. (12), Doğan et al. (13). However, there is no description of the treatment for this disease. Here, we report the first case of DE-induced BCS that was successfully resolved by diaphragmatic plication (DP), a minimally invasive surgery. This case was reported in accordance with the CARE Guidelines (14).

## Case report

**FIGURE 1:**
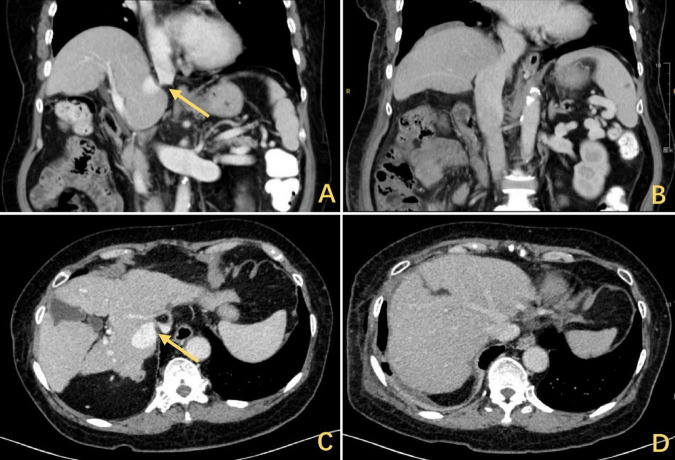
The CT scan showed a DE with hepatic elevation leading to a reduction in the angle of confluence between the HV and the IVC and obstruction of the hepatic outflow tract. The arrows highlighted in yellow indicate the stenosis, **(A)** coronal and **(C)** axial. Release of outflow tract obstruction after DP, **(B)** coronal and **(D)** axial. CT, computed tomography; DE, diaphragmatic eventration; HV, hepatic vein; IVC, inferior vena cava; DP, diaphragmatic plication.

**FIGURE 2:**
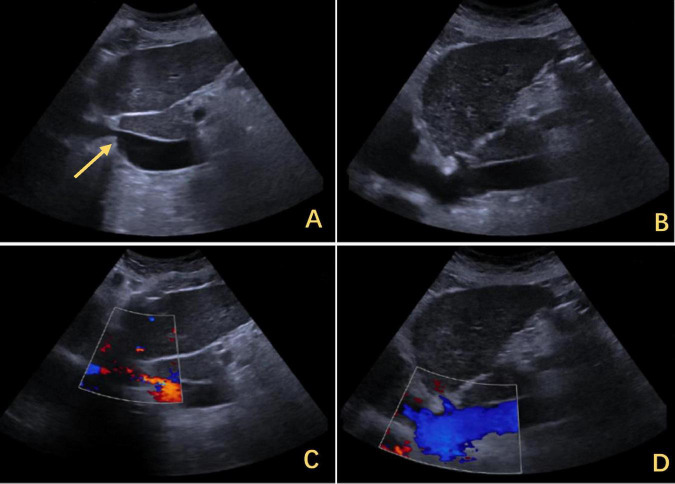
Ultrasound findings of IVC stenosis with outflow obstruction. **(A)** The IVC was approximately 0.33 cm wide, with its lower segment expanding to 2.8 cm. The arrows highlighted in yellow indicate stenosis. **(C)** The flow increased to 0.134 m per second. **(B)** After the release of the outflow tract obstruction after DP, the diameter of the IVC increased to 1.2 cm. **(D)** The flow rate returned to normal. IVC, inferior vena cava; DP, diaphragmatic plication.

The patient was a 72 years-old Chinese woman who was admitted with chronic abdominal pain lasting more than 30 years and recent reduced directionality lasting 2 weeks. A total of 15 days prior to presentation, she experienced blurred consciousness upon waking up in the morning. Cranial computed tomography (CT) imaging and magnetic resonance imaging (MRI) were performed at another hospital to exclude cerebrovascular disease and other abnormalities, and a scale-based assessment suggested disorientation. The patient’s blood ammonia level reached 90.2 μmol/L, while her liver and kidney function were normal. After treatment with oral lactulose and mentholated ornithine preparations, improvements in orientation and consciousness were observed. The patient had a history of type II diabetes mellitus and was taking metformin and glimepiride regularly, achieving good glycemic control. No other significant findings were noted in terms of family history, psychosocial history, or lifestyle history. On physical examination, the vital signs were normal, the skin and sclera were not yellowed, no mass was palpable in the upper abdomen, no tenderness was found in the abdomen, and Murphy’s sign was negative. After consulting our hospital, the following blood laboratory indices were measured: white blood cell (WBC) count, 3.69 × 10^9^/L; hemoglobin (HBG), 105 g/L; alanine transaminase (ALT), 24.6 U/L; aspartate aminotransferase (AST), 43.9 U/L; and total bilirubin (TBIL), 33.7 mmol/L. Contrast-enhanced CT imaging revealed narrowing of the IVC above the HV confluence, cirrhosis, dilation of the main portal trunk to 13 mm, a tortuous splenic vein and esophagogastric fundic varices (Figure 1). Abdominal ultrasound revealed a constriction of the IVC (at the opening of the HV) approximately 0.33 cm in width, an increase in flow velocity to 134 cm per second (Figure 2C) and a dilatation of the IVC at its lower segment to 2.8 cm (Figure 2A). Combined with the patient’s CT and ultrasound assessment of IVC morphology, it was evident that the hepatic outflow tract was obstructed, thus confirming the diagnosis of BCS.

**FIGURE 3:**
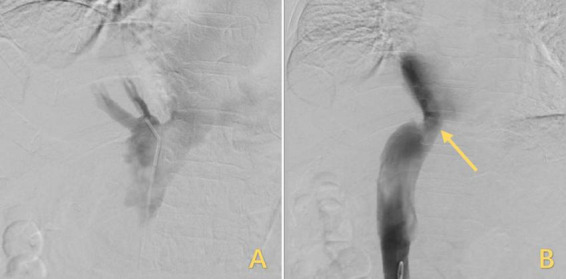
Treatment for IVC stenosis with interventional balloon dilatation. **(A)** The inflated balloon was used to open the narrowed IVC. **(B)** After the balloon was withdrawn, the IVC returned to its original shape, but the stenosis remained unrelieved, and the arrows highlighted in yellow indicate the stenosis. IVC, inferior vena cava.

**FIGURE 4:**
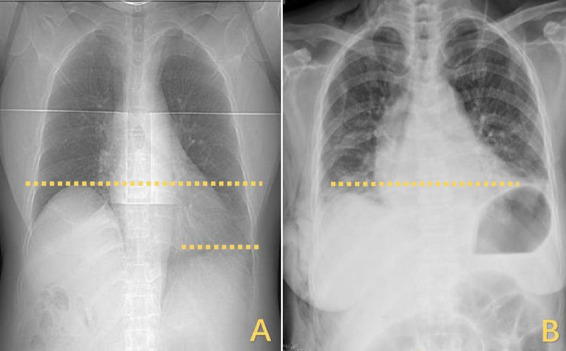
Changes in the horizontal position of the right diaphragm before and after DP. **(A)** The right diaphragm was significantly raised compared to the left diaphragm, which was elevated by approximately three vertebral levels. **(B)** Postoperatively, both diaphragms returned to the same level. DP, diaphragmatic plication.

**FIGURE 5:**
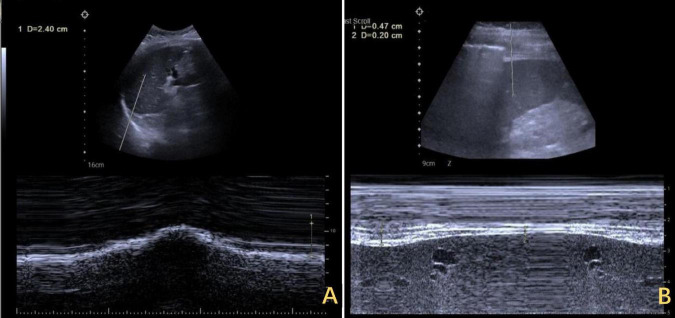
Ultrasound assessment of bilateral diaphragm amplitude. **(A)** The left diaphragm showed an amplitude of 2.4 cm at end of inspiration. **(B)** The right diaphragm exhibited minimal amplitude throughout the respiratory cycle, ranging from 0.20 to 0.47 cm.

However, the patient’s treatment process was not very smooth, and the patient experienced twists and turns. The treatment of BCS began with an interventional approach using a balloon to dilate and shape the stenotic IVC. The patency of the IVC was restored after balloon inflation (Figure 3A), but the original stenosis returned immediately after balloon withdrawal, and there was no change in the internal diameter of the stenosis (Figure 3B). There was no benefit from three rounds of balloon inflation. Postoperative chest radiographs showed a marked elevation of the right diaphragm in contrast to the left diaphragm, which was elevated by approximately three vertebral levels, consistent with a diagnosis of DE (Figure 4A). Subsequently, ultrasound examination of diaphragmatic mobility revealed that during calm breathing, the right diaphragm amplitude was 0.20–0.47 cm and did not change significantly; the left diaphragm amplitude was 2.4 cm; which was consistent with the diagnosis of right DE (Figure 5). Tumors of thoracic and mediastinal origin and those caused by inflammation were ruled out by positron emission tomography/computed tomography (PET/CT). Based on these findings, the CT images were further reviewed. It was suggested that the elevation of the diaphragm led to liver transposition and torsion of the HV at the confluence of the IVC. This approach helped to clarify the distortion of the IVC despite several interventional balloon dilatations due to the external force exerted by liver traction. To prevent further elevation of the diaphragm from worsening the obstruction, DP was recommended by thoracic surgeons. However, the efficacy of this procedure for treating BCS is inconclusive and has not been reported in the literature. Nonetheless, DP minimally invasive, and even if it is ineffective, it does not increase the difficulty of open surgery.

**FIGURE 6:**
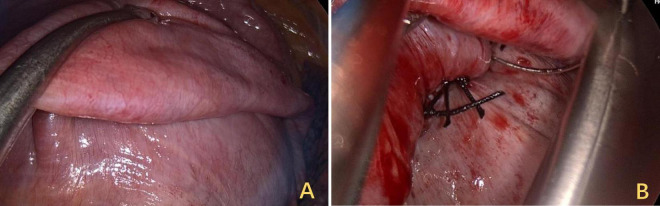
Surgical field view of trans-thoracoscopic DP. **(A)** Restoring tension by folding the diaphragm onto a flat surface. **(B)** At the base of the folded segment, sutures were used for continuous suturing and fixation. DP, diaphragmatic plication.

A 3 cm incision was made at the right mid-axillary line, intercostal space five, and the pleural cavity was entered by dissecting through the layers. Intraoperatively, thoracoscopy was performed for exploration, revealing no pleural effusion or pleural nodules, and no pleural adhesions were observed. Electrical stimulation of the phrenic nerves resulted in a weak diaphragmatic contraction response. Pressing down on the diaphragm was attempted, followed by esophageal ultrasound, which revealed that the width of the IVC increased to 14 mm. The tendon plate area of the diaphragm was lifted with oval forceps (Figure 6A), the root was sutured, and the free discarded diaphragm was fixed (Figure 6B). A thoracic close drainage was preplaced, and after adequate lung reinflation and air evacuation, the drainage was removed.

**FIGURE 7:**
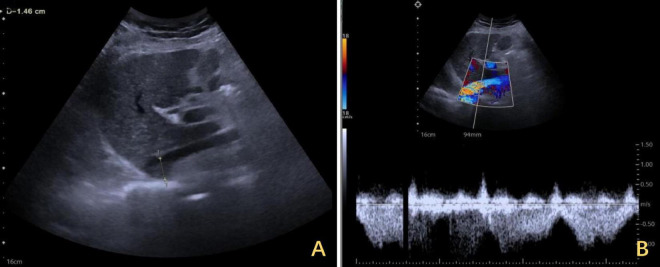
One-year follow-up ultrasound of the IVC after DP. **(A)** The maximum diameter of the IVC was 1.46 cm at its widest segment. **(B)** The IVC exhibited a mean flow velocity of 0.5 m per second, with a peak velocity of approximately 1 m per second. IVC, inferior vena cava; DP, diaphragmatic plication.

**FIGURE 8:**
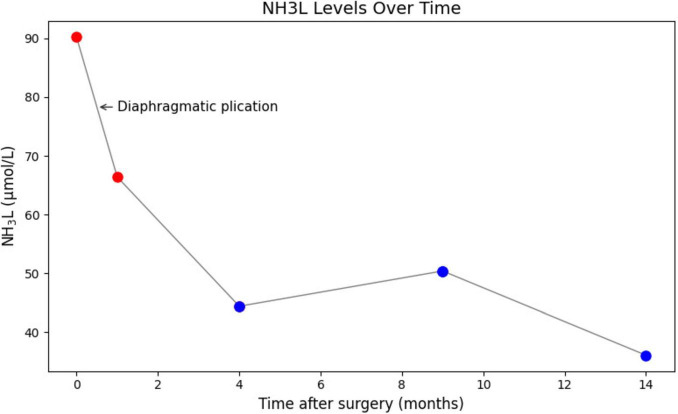
Serial blood ammonia monitoring following DP. Blood ammonia levels decreased immediately postoperatively and remained within normal range. DP, diaphragmatic plication.

The right diaphragm dropped to almost the same level as the left diaphragm on the third day after DP (Figure 4B). The patency of the IVC stenosis was restored with by increasing the internal diameter to 1.2 cm (Figure 2B). And the flow rate returned to normal (Figure 2D). The patient’s blood ammonia level decreased to 66.4 μmol/L. She had a thoracic close drainage removed on the third postoperative day, had no postoperative complications, and was discharged on the fourth postoperative day. At 1 year postoperative follow-up, ultrasonographic evaluation of IVC revealed a maximum diameter of 1.46 cm, with a mean flow velocity of 0.5 m per second and peak velocity of approximately 1 m per second (Figure 7). Serial blood ammonia monitoring demonstrated persistently normal levels throughout the follow-up period (Figure 8).

## Discussion

Only three cases of DE-induced BCS have been reported in the literature. The diagnosis of these patients is challenging and can only be deduced by fully combining the patients’ imaging data and clinical symptoms. The case described in this article was diagnosed only after a review of judgment, following several failed interventions. Therefore, this disease is rare and often misdiagnosed, leading to delayed or ineffective treatment.

The key to resolving secondary BCS is to remove the cause of the obstruction, e.g., the removal of the solid tumor and the tumor thrombus and the closure of the hernia ring to repair the diaphragmatic hernia. In contrast, the etiology of BCS does not lend itself to conventional surgical solutions. Diaphragmatic paralysis resulting in DE causes a change in the anatomical position of the liver, resulting in a corresponding increase of 2–3 vertebral levels. Additionally, the angle at which the HE converges into the IVC changes from an obtuse to an acute angle (15). This process is accompanied by a compensatory increase in hepatic outflow through short veins that originate from the central and caudate lobes of the liver and connect directly to the IVC (16). The central hepatic lobe becomes hypertrophied, and the lobe in which outflow tracts drain through the obstructing hepatic vein becomes atrophied (17). This results in changes in the center of gravity of the liver that contribute to its axial displacement, which is centered on the IVC. This displacement further reduces the angle of HV-IVC convergence and exacerbates outflow tract obstruction (15).

In conventional surgery, it is assumed that resecting part of the liver and the abnormal diaphragm, and simultaneously repairing the diaphragmatic defect with a artificial patch, will restore the anatomical position and thereby resolve the confluence obstruction (18). However, this approach is highly invasive and involves permanent implantation of artificial materials, which have a limited lifespan. Importantly, no effective treatments were available for the three previously reported cases (12, 13). In our case, the treatment was innovative, and minimally invasive thoracoscopic surgery can achieve the same efficacy as conventional treatments by lowering the plane of the liver by folding the diaphragm of the inflated segment. Based on accurate diagnostic reasoning, a suitable minimally invasive treatment was chosen, resulting in a satisfactory therapeutic outcome.

Congenital weakness of the diaphragm is the most common cause of DE (6). The diaphragm is attached to the thoracic ribs and vertebrae. The prevalence of DE in the population is less than 0.05%, and it is more common on the left side than on the right (19). The first report of using DP to treat DE was published in 1923 by Morrison et al. (20). Since then, the safety and efficacy of DP have been recognized, and thus, it has been widely practiced. Beshay et al. (21) reviewed the postoperative lung function of 134 patients who underwent DP and found that the procedure was effective in reducing debilitating dyspnea and improving lung function in patients suffering from diaphragm eventration. Groth et al. (22) followed up with their patients after DP by collecting questionnaire responses and reported significant short-term and midterm improvements in respiratory quality of life and pulmonary function test results. A retrospective study by Kocher et al. (23) reported that performing minimally invasive DP not only significantly improves respiratory symptoms but also simplifies the procedure, increases efficiency, and ensures perioperative safety. The diaphragm can be approached from the thorax or the abdomen. Either approach may be performed with open or minimally invasive techniques. The only goal of diaphragm plication is to treat dyspnea; hence, operative intervention is indicated exclusively for patients who are symptomatic (19). Studies suggest that the procedure leads to an improvement in vital capacity, forced expiratory volume, and overall lung function, particularly in patients with diaphragmatic paralysis due to phrenic nerve injury or other causes of diaphragm dysfunction (24–27). While the recurrence of diaphragmatic dysfunction is uncommon, it can occur in some cases, particularly if the underlying cause is not fully addressed (e.g., ongoing phrenic nerve damage). Long-term failure rates are generally low, but it remains a potential concern for a subset of patients (28).

In this case study, DP reversed the liver dysfunction caused by BCS and even alleviated the need for liver transplantation. Surgical indications for DP have expanded, thus providing a new option for treating such patients.

## Conclusion

A subset of BCS patients require CT, MRI and ultrasound to determine IVC morphology and alignment after primary etiology has been ruled out. DH and DE are extremely rare causes of BCS. DP can be an effective and minimally invasive treatment for BCS if it is clearly diagnosed. Future multicenter studies involving larger patient cohorts will be essential to confirm these findings and establish stronger evidence-based medical support.

## Data Availability

The original contributions presented in this study are included in this article/supplementary material, further inquiries can be directed to the corresponding author.

## References

[1] Martens P Nevens F. Budd-Chiari syndrome. *United European Gastroenterol J.* (2015) 3:489–500. 10.1177/2050640615582293 26668741 PMC4669515

[2] Menon K Shah V Kamath P. The Budd-chiari syndrome. *N Engl J Med.* (2004) 350:578–85. 10.1056/NEJMra020282 14762185

[3] Valla D. Primary Budd-Chiari syndrome. *J Hepatol.* (2009) 50:195–203. 10.1016/j.jhep.2008.10.007 19012988

[4] Ludwig J Hashimoto E McGill D van Heerden J. Classification of hepatic venous outflow obstruction: Ambiguous terminology of the Budd-Chiari syndrome. *Mayo Clin Proc.* (1990) 65:51–5. 10.1016/s0025-6196(12)62109-0 2296212

[5] Neelamraju Lakshmi H Saini D Om P Bagree R. A ventral incisional hernia with herniation of the left hepatic lobe and review of the literature. *BMJ Case Rep.* (2015) 2015:bcr2014207162. 10.1136/bcr-2014-207162 25631758 PMC4322246

[6] Deslauriers J. Eventration of the diaphragm. *Chest Surg Clin N Am.* (1998) 8:315–30. 9619307

[7] Schumpelick V Steinau G Schlüper I Prescher A. Surgical embryology and anatomy of the diaphragm with surgical applications. *Surg Clin North Am.* (2000) 80:213–39. 10.1016/s0039-6109(05)70403-5 10685150

[8] Thomas T. Congenital eventration of the diaphragm. *Ann Thorac Surg.* (1970) 10:180–92. 10.1016/s0003-4975(10)65584-1 4913762

[9] Efthimiou J Butler J Woodham C Benson M Westaby S. Diaphragm paralysis following cardiac surgery: Role of phrenic nerve cold injury. *Ann Thorac Surg.* (1991) 52:1005–8. 10.1016/0003-4975(91)91268-z 1929616

[10] Markand O Moorthy S Mahomed Y King R Brown J. Postoperative phrenic nerve palsy in patients with open-heart surgery. *Ann Thorac Surg.* (1985) 39:68–73. 10.1016/s0003-4975(10)62524-6 3966838

[11] Thomas T. Nonparalytic eventration of the diaphragm. *J Thorac Cardiovasc Surg.* (1968) 55:586–93. 10.1016/S0022-5223(19)42949-8 5644518

[12] Saujani S Rahman S Fox B. Budd-Chiari syndrome due to right hepatic lobe herniation: CT image findings of two rare clinical conditions. *BJR Case Rep.* (2017) 3:20160133. 10.1259/bjrcr.20160133 30363244 PMC6159189

[13] Doğan U Ozdemir K Paksoy Y Gök H. Dynamic obstruction of inferior vena cava flow caused by right-sided diaphragmatic elevation. *Anadolu Kardiyol Derg.* (2010) 10:E19–20. 10.5152/akd.2010.152 20929685

[14] Riley D Barber M Kienle G Aronson J von Schoen-Angerer T Tugwell P CARE guidelines for case reports: Explanation and elaboration document. *J Clin Epidemiol.* (2017) 89:218–35. 10.1016/j.jclinepi.2017.04.026 28529185

[15] Kim P Mitchell D Outwater E. Budd-Chiari syndrome: Hepatic venous obstruction by an elevated diaphragm. *Abdom Imaging.* (1999) 24:267–71. 10.1007/s002619900493 10227891

[16] Tilanus H. Budd-Chiari syndrome. *Br J Surg.* (1995) 82:1023–30. 10.1002/bjs.1800820807 7648141

[17] Mitchell D Nazarian L. Hepatic vascular diseases: CT and MRI. *Semin Ultrasound CT MR.* (1995) 16:49–68. 10.1016/0887-2171(95)90014-4 7718282

[18] Wcislo K Hall C Abbassi-Ghadi N. Acute Budd-Chiari syndrome caused by inferior vena cava compression from a congenital diaphragmatic hernia. *Ann R Coll Surg Engl.* (2020) 102:e202–4. 10.1308/rcsann.2020.0106 32538105 PMC7538725

[19] Groth S Andrade R. Diaphragmatic eventration. *Thorac Surg Clin.* (2009) 19:511–9. 10.1016/j.thorsurg.2009.08.003 20112634

[20] Ricoy J Rodríguez-Núñez N Álvarez-Dobaño J Toubes M Riveiro V Valdés L. Diaphragmatic dysfunction. *Pulmonology.* (2019) 25:223–35. 10.1016/j.pulmoe.2018.10.008 30509855

[21] Beshay M Abdel Bary M Kösek V Vordemvenne T Mertzlufft F Schulte Am Esch J. Minimally-invasive diaphragmatic plication in patients with unilateral diaphragmatic paralysis. *J Clin Med.* (2023) 12:5301. 10.3390/jcm12165301 37629343 PMC10455218

[22] Groth S Rueth N Kast T D’Cunha J Kelly R Maddaus M Laparoscopic diaphragmatic plication for diaphragmatic paralysis and eventration: An objective evaluation of short-term and midterm results. *J Thorac Cardiovasc Surg.* (2010) 139:1452–6. 10.1016/j.jtcvs.2009.10.020 20080267

[23] Kocher G Zehnder A Schmid R. Completely thoracoscopic diaphragmatic plication. *World J Surg.* (2017) 41:1019–22. 10.1007/s00268-016-3789-2 27822722

[24] Versteegh M Braun J Voigt P Bosman D Stolk J Rabe K Diaphragm plication in adult patients with diaphragm paralysis leads to long-term improvement of pulmonary function and level of dyspnea. *Eur J Cardiothorac Surg.* (2007) 32:449–56. 10.1016/j.ejcts.2007.05.031 17658265

[25] Kaufman M Elkwood A Brown D Cece J Martins C Bauer T Long-term follow-up after phrenic nerve reconstruction for diaphragmatic paralysis: A review of 180 patients. *J Reconstr Microsurg.* (2017) 33:63–9. 10.1055/s-0036-1588018 27665114

[26] Freeman R Van Woerkom J Vyverberg A Ascioti A. Long-term follow-up of the functional and physiologic results of diaphragm plication in adults with unilateral diaphragm paralysis. *Ann Thorac Surg.* (2009) 88:1112–7. 10.1016/j.athoracsur.2009.05.027 19766791

[27] Hunt A Stuart C Gergen A Bang T Reihman A Helmkamp L Long-term patient-reported symptom improvement and quality of life after transthoracic diaphragm plication in adults. *J Am Coll Surg.* (2023) 237:533–44. 10.1097/XCS.0000000000000762 37194947

[28] Heng L Alzahrani K Montalva L Podevin G Schmitt F. Congenital diaphragmatic eventration: Should we maintain surgical treatment? A retrospective multicentric cohort study. *J Pediatr Surg.* (2025) 60:161991. 10.1016/j.jpedsurg.2024.161991 39442326

